# The Effect of Superparamagnetic Iron Oxide Nanoparticle Surface Charge on Antigen Cross-Presentation

**DOI:** 10.1186/s11671-017-1828-z

**Published:** 2017-01-19

**Authors:** Yongbin Mou, Yun Xing, Hongyan Ren, Zhihua Cui, Yu Zhang, Guangjie Yu, Walter J. Urba, Qingang Hu, Hongming Hu

**Affiliations:** 1 Nanjing Stomatological Hospital, Medical School of Nanjing University, 30#, Zhongyang Road, Nanjing, 210008 People’s Republic of China; 2 Laboratory of Cancer Immunobiology, Robert W. Franz Cancer Research Center, Earle A. Chiles Research Institute, Providence Cancer Center, Portland, OR USA; 3 Minigene Pharmacy Laboratory, School of Life Science and Technology, China Pharmaceutical University, Nanjing, People’s Republic of China; 4 Medical School, Southeast University, Nanjing, People’s Republic of China; 5 State Key Laboratory of Molecule and Bimolecular Electronics, Jiangsu Provincial Laboratory for Biomaterials and Devices, Southeast University, Nanjing, People’s Republic of China; 6 Cancer Research, Robert W. Franz Cancer Research Center, Earle A. Chiles Research Institute, Providence Cancer Center, 4805 NE Glisan Street, Portland, OR 97213 USA

**Keywords:** Superparamagnetic iron oxide, Dendritic cells, Adjuvant, Antigen cross-presentation

## Abstract

Magnetic nanoparticles (NPs) of superparamagnetic iron oxide (SPIO) have been explored for different kinds of applications in biomedicine, mechanics, and information. Here, we explored the synthetic SPIO NPs as an adjuvant on antigen cross-presentation ability by enhancing the intracellular delivery of antigens into antigen presenting cells (APCs). Particles with different chemical modifications and surface charges were used to study the mechanism of action of antigen delivery. Specifically, two types of magnetic NPs, γFe_2_O_3_/APTS (3-aminopropyltrimethoxysilane) NPs and γFe_2_O_3_/DMSA (meso-2, 3-Dimercaptosuccinic acid) NPs, with the same crystal structure, magnetic properties, and size distribution were prepared. Then, the promotion of T-cell activation via dendritic cells (DCs) was compared among different charged antigen coated NPs. Moreover, the activation of the autophagy, cytosolic delivery of the antigens, and antigen degradation mediated by the proteasome and lysosome were measured. Our results indicated that positive charged γFe_2_O_3_/APTS NPs, but not negative charged γFe_2_O_3_/DMSA NPs, enhanced the cross-presentation ability of DCs. Increased cross-presentation ability induced by γFe_2_O_3_/APTS NPs was associated with increased cytosolic antigen delivery. On the contrary, γFe_2_O_3_/DMSA NPs was associated with rapid autophagy. Overall, our results suggest that antigen delivered in cytoplasm induced by positive charged particles is beneficial for antigen cross-presentation and T-cell activation. NPs modified with different chemistries exhibit diverse biological properties and differ greatly in their adjuvant potentials. Thus, it should be carefully considered many different effects of NPs to design effective and safe adjuvants.

## Background

It is taken for granted that adjuvants are key components of vaccines and play an important role in a strong immune response. New generation of adjuvants, including toll like receptors (TLRs) activating agonists [1, 2], pH-responsive polymeric NPs [3], gene-silencing complexes [4, 5] and so on, is being developed to induce a more effective cell-mediated immune response against tumor, intracellular bacteria or viruses. As an important approach, biomedical applications of nanomaterials are usually related to the immune system and deeply involved in health and disease. Therefore, the interactions of adjuvant NPs with the immune system and its potential effects and implications are key questions that should be answered to take full advantage of such approaches.

Due to the multifunctional properties of SPIO NPs, such as small particle size, superparamagnetism, and biocompatibility, there are many different kinds of these particle applications in biomedicine, mechanics, and information. In biomedicine, many studies demonstrate SPIO NP applications in diagnosis and treatment, such as a contrast enhancement agent for magnetic resonance imaging, drug carriers for a drug delivery system, generator of heat for tumor hyperthermia and detoxification of biological fluids [6–8]. Recent reports show that NPs, such as Al_2_O_3_, TiO_2_, ZnO, or SiO_2_ NPs, can be used as an antigen delivery carrier to absorb and bring more antigens into APCs, which are slowly released into the APCs, resulting in an enhanced the immune response [9–11]. As the mental adjuvant NPs, γFe_2_O_3_ NPs with positive charge characteristic could be absorbed by protein with negative charge, which may has the similar characteristic to promote the immune response accompany with their functions of cells labeling and tracking [12, 13].

In the development of adaptive immunity to tumours and most infectious pathogens, professional APCs, such as DCs, are capable of presenting exogenous antigens to CD8 positive cytotoxic T lymphocytes in a pivotal process, which is known as antigen cross-presentation. Cross-presentation is a sequential, multi-step process that involves antigen internalization, protein degradation, and loading of antigen-derived peptides into major histocompatibility complex class I (MHC-I) molecules of APCs. The classical and effective pathway of antigen cross-presentation has been studied in detail [14]. Cytosolic and nuclear antigens are degraded into peptides by the proteasome and transported from the cytosol into the endoplasmic reticulum (ER) by the protein transporter TAP. These peptides are then loaded onto newly synthesized MHC-I, and these complexes released from the ER are transported to the cell surface via the Golgi [14]. Up to date, some antigen-nanoparticle complexes could enhance antigen cross-priming of cytotoxic T lymphocytes, but the mechanisms are still poorly understood.

In this study, we sought to investigate that synthetic γFe_2_O_3_ NPs modified with opposite charged polymers have different functions as an adjuvant property for their ability to promote cell-mediated immunity. Furthermore, we presented a novel direction of antigen carried by NPs modified with different chemistries exhibiting diverse delivery passway in their adjuvant potentials.

## Methods

### Preparation of the SPIO

γFe_2_O_3_ NPs were prepared according to the method we described previously [12]. Briefly, a solution of FeCl_3_ and FeSO_4_ (molar ratio 2:1) was prepared under N_2_ protection and stirred vigorously at room temperature for 30 min. The resulting Fe_3_O_4_ NPs were obtained and washed immediately with distilled water five times by magnetic separation. The final precipitates were dispersed in distilled water at a concentration of 3 mg/ml and a pH of 3.0. Finally, the precipitates were oxidized into brown γFe_2_O_3_ NPs by aeration at 95 °C, γFe_2_O_3_ NPs were then coated with DMSA and APTS according to the process described in literature [15]. Briefly, DMSA aqueous solution was added to 100 ml of γFe_2_O_3_ NPs solution (molar ratio of DMSA and [Fe] was 1:40). The reaction was carried out for 4 h with continuous stirring. γFe_2_O_3_ NPs (2 mg/ml) was stirred at a rate of 500 rpm at 50 °C. APTS (APTS and [Fe] was 0.2:1) was added and stirred for 5 h. The precipitate was separated with a permanent magnet, washed with deionized water and, at the same time, placed in an ultra sonicator. Finally, the NPs samples were dried into powder at room temperature under vacuum.

### Cell Lines

The Mutu DC cell line, named for murine tumor and kindly provided by Prof. Hans Acha-Orbea (University of Lausanne, Switzerland), was originated from spleen tumors in CD11c:SV40LgT-transgenic C57BL/6 mice [16]. B_3_Z cell line, a CD8^+^ T cell hybridoma expressing LacZ gene when its T cell receptor engages an OVA_258–265_ epitope in the context of H-2K^b^ MHC class I molecule, was a gift of Prof. Nilabh Shastri (University of California, Berkeley, CA) [17].

### Electron Microscopy Imaging and Surface Charges of Particles

Mutu DCs (2 × 10^6^) were incubated with the particles, either γFe_2_O_3_/APTS or γFe_2_O_3_/DMSA (50 μg/mL) for 6 h, which were cocultured with OVA protein (10 μg/ml, Sigma-Aldrich) for 1 h before cocultured with cells. After being fixed, ultra-thin sections (90 nm) were cut and stained with 1% osmium tetroxide and 0.8% potassium ferrocyanide in 100 mM sodium cacodylate buffer for 2 h. After being rinsed, stained, and dehydrated, samples were then infiltrated with a 1:1 mix of acetone and Epon 812 (EMS cat#14120) overnight with rotation. After this incubation step, the 1:1 mix was replaced with Epon 812 and allotted time to polymerize overnight at 60 °C. Thin sections obtained from the block face were imaged at 80 kV on a FEI-Tecnai 12 system interfaced to a digital camera and analyzed with the associated software (Advanced Microscopy Techniques, Danvers, MA).

The surface charges of NPs with or without OVA protein were measured by a zeta potential assay [15]. The particles of γFe_2_O_3_/APTS, γFe_2_O_3_/DMSA, OVA-γFe_2_O_3_/APTS, and OVA-γFe_2_O_3_/DMSA were prepared with different pH values from 3 to 8 and the concentrations of NPs and OVA were adjusted to 25 and 2.5 μg/ml, respectively. The zeta potentials of the samples were measured using a zetasizer Nano ZS90 potential analyzer (Malvern, UK).

### In Vitro Antigen Cross-Presentation Assay

Mutu DCs and transporter associated with antigen processing 1 (TAP1) knockout DCs (2 × 10^4^) were pulsed with γFe_2_O_3_ NPs coated with different polymers with or without proteasomal inhibitor, Bortezomib/Velcade (200nM, Millennium), and lysosomal inhibitor, NH_4_Cl (20 μM, Sigma-Aldrich), which incubated with OVA protein (10 μg/ml, Sigma-Aldrich) for 1 h at room temperature. After 6 h, DCs were cocultured with B_3_Z (2 × 10^5^) overnight. The B_3_Z cells response was measured as β-galactosidase activity induced upon ligand recognition. The β-galactosidase activity was measured by the sample’s absorbance at 595 nm, the absorbance of the cleavage product of Chlorophenol Red-β-D-Galactopyranoside (CPRG, Sigma-Aldrich). The assay of antigen cross-presentation was called CPRG in this study.

### Western Blot and Bicinchoninic Acid (BCA) Analysis

In order to detect OVA expression in DCs, cells (5 × 10^6^) were pulsed with γFe_2_O_3_/APTS, γFe_2_O_3_/DMSA (100 μg/ml) cocultured with OVA (10 μg/ml) for 6 hours, and then cytosol collected after treated by Perfringolysin O (PFO, 100 ng/ml) at 37 °C for 30 min. These samples, concentrated by Methanal (Fisher Scientific) and Chloroform (Sigma-Aldrich), were detected by chemiluminescent reagents (Bio-Rad) and then incubated with anti-OVA (Sigma-Aldrich) and secondary antibody (Thermo Scientific) for Western Blot analysis.

Mutu DCs (5 × 10^5^) were pulsed with γFe_2_O_3_/APTS and γFe_2_O_3_/DMSA (100 μg/ml) cocultured with OVA (10 μg/ml) at 37 °C for different time points. Samples of cell total lysates were incubated with the antibody of anti-OVA and anti-LC3 proteins (Santa cruz) at 4 °C overnight and secondary antibody (Thermo Scientific) labeled with horseradish peroxidase for another one hour in the following morning.

To determine the amount of OVA protein absorbed by NPs, OVA protein (10 and 100 μg) was cocultured with γFe_2_O_3_/APTS NPs or γFe_2_O_3_/DMSA NPs (100 μg) for one hours at room temperature. A microplate BCA assay kit (Pierce, Rockford, IL, USA) was used to measure the total protein content of OVA protein absorbed by NPs according to manufacturer’s instructions. Bovine serum albumin (BSA) provided in the kit was used as the standard curve, and absorbance was read at 560 nm.

### Statistical Analysis

Data were analyzed using the Statistical Package for Social Science (version 13.0, SPSS Inc., Chicago, IL, USA). Results were expressed as means ± standard deviation. Differences between control and test groups were assessed by one-way analysis of variance, two-tailed Student’s *t* tests, and double factor analysis of variance. The level of statistical significance was set at *P* < 0.05.

## Results

### Characterization of γFe_2_O_3_ NPs with Different Coatings

**Fig. 1:**
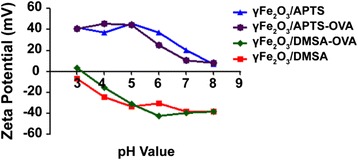
pH-dependent zeta potential curves of γFe_2_O_3_ NPs coated with different charged molecules

To determine the surface charges of NPs, we first measured the zeta potentials. The γFe_2_O_3_ NPs were coated with DMSA or APTS and with or without OVA protein. The pH values of the medias that were used to suspend different γFe_2_O_3_ NPs were titrated to the levels from 3 to 8. Zeta potentials of γFe_2_O_3_/APTS NPs with or without OVA protein showed positive charge characteristics, which were not influenced by the pH values of the suspension medium. On the contrary, γFe_2_O_3_/DMSA NPs showed negative charge characteristics with the exception of point of zero charge when the pH value was 3 (Fig. 1). Therefore, our data infer that γFe_2_O_3_/APTS NPs will stay positive charged when the pH is below 5, but its charge will reduce as the pH value is higher than 5. On the contrary, the potentials of γFe_2_O_3_/DMSA NPs keeps negative when the pH values were between 5 and 8 With pH values declining from 5 to 3, the potentials gradually close in on the isoelectric point (IP).

### γFe_2_O_3_ NPs Activated Murine DCs Cross-Presentation

**Fig. 2:**
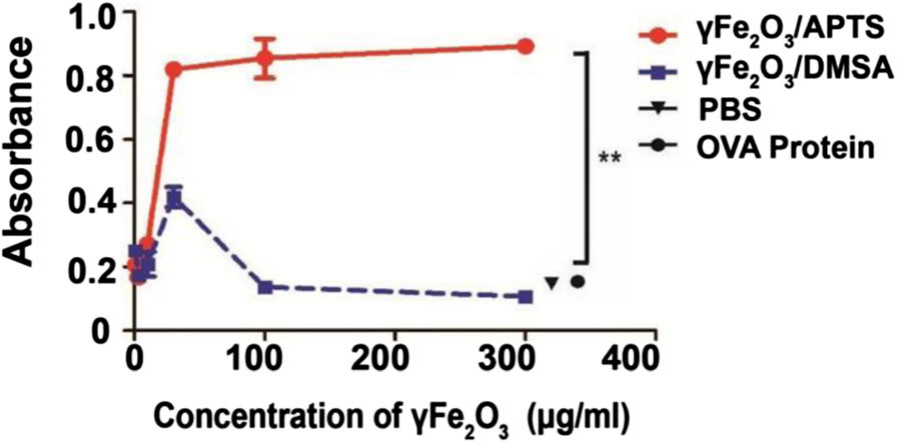
γFe_2_O_3_ NPs with different charge molecules active Mutu DCs cross-presentation

To further investigate the effect of surface on the T-cell activation in a murine system, γFe_2_O_3_/APTS and γFe_2_O_3_/DMSA NPs were incubated with OVA protein at different concentrations for 1 h at room temperature and loaded into Mutu DCs for 6 h. Five dose ratios of γFe_2_O_3_ NPs were adopted in this study, which were 3, 10, 30, 100, and 300 μg/ml. Then B_3_Z cells were cocultured with Mutu DCs for another 12 h. The degree of T-cell activation was determined by measuring the production of beta-galactosidase with CPRG assay as the colorimetric substrate. As shown in Fig. 2, we observed that γFe_2_O_3_/APTS coated with 30–300 μg/ml OVA protein yielded a sufficient response of antigen cross-presentation and there were no significant differences between these concentrations. On the contrary, γFe_2_O_3_/DMSA NPs had no significant effect on the cross- presentation. Meanwhile, the same doses of OVA protein alone were also not cross-presented to T cells by Mutu DCs.

### Cross-Presentation of OVA Protein Dependents on Proteasome TAP1 Pathway

**Fig. 3:**
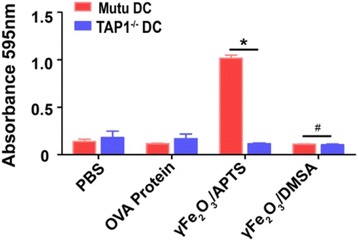
Cross-presentation of OVA protein by DCs through TAP1 passway

To investigate the mechanism of OVA protein cross-presentation by DCs, murine BMDCs were generated from TAP1 knockout mice and cocultured with γFe_2_O_3_/APTS and γFe_2_O_3_/DMSA coated with OVA protein before they were used to stimulate B_3_Z T cells. The CPRG results showed that T cells incubated with TAP1 knockout BMDCs had no significant response to B_3_Z cells. (Fig. 3).

**Fig. 4:**
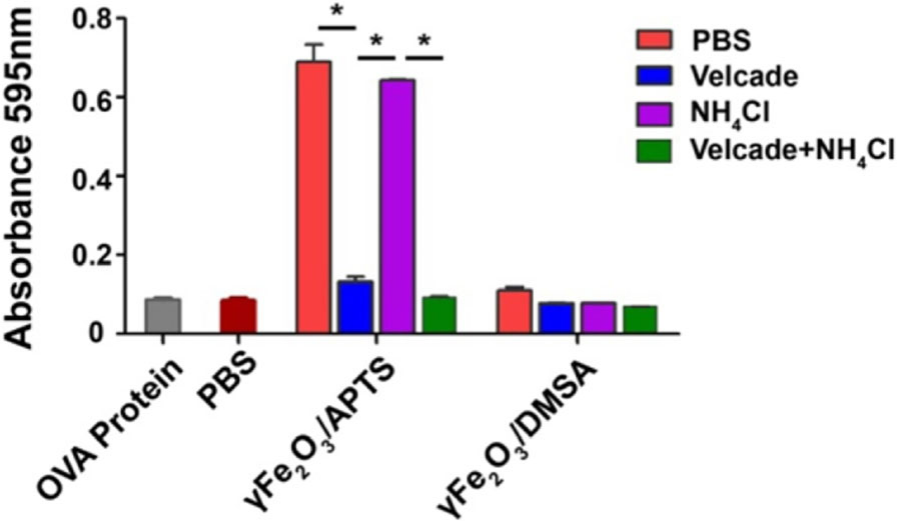
Cross-presentation of OVA protein through proteasome in Mutu DCs

To further assess whether cross-presentation of OVA protein with NPs requires proteasome or lysosome degradation for presentation, we used velcade and NH_4_Cl, which are highly specific inhibitors of the proteasome and lysosome [18]. After incubated with γFe_2_O_3_ NPs coated OVA protein and different inhibitors for 6 h, DCs cross-presentation capacity was strongly inhibited by velcade but not by NH_4_Cl (Fig. 4). These results showed that cross-presentation mediated by γFe_2_O_3_ NPs was proteasome and TAP1 dependent, but lysosome-independent.

### Location of NPs with Opposite Charges in DCs Under TEM

**Fig. 5:**
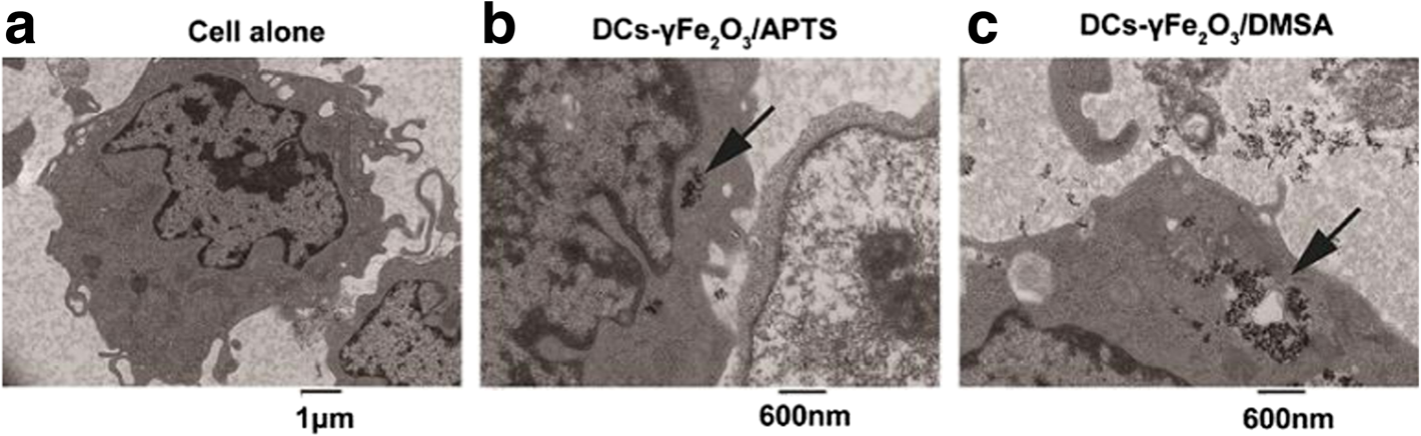
**a** DCs, which were without γFe O NPs labeling, had round shape and the cytoplasm was uniform. The scale bar represents 1μm. DCs labeled with γFe O /APTS (**b**) and γFe O /DMSA (**c**) were imaged by the magnification of 600 nm

The ultrastructure of DCs labeled with γFe_2_O_3_ NPs with opposite charged polymers and protein were observed by using transmission electron microscopy (TEM). As shown in Fig. 5, DCs treated with γFe_2_O_3_ NPs displayed electron-dense NPs compared to untreated cells, which displayed numerous γFe_2_O_3_ NPs huddled together in the cytoplasm.

The γFe_2_O_3_ NPs with positive APTS were either swallowed by endosomes or remained free in the cytoplasm (Fig. 5b). On the contrary, more γFe_2_O_3_ NPs with the negative polymer, DMSA, were observed in the cytoplasm of DCs and almost all the particles were surrounded by double or more layered membrane structures that resembled autolysosomes could be seen. (Fig. 5c).

### Induction of Autophagy by γFe_2_O_3_ NPs with Different Charged Polymers

**Fig. 6:**
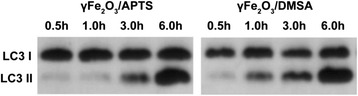
Autophagy in Mutu DCs pulsed with γFe_2_O_3_ NPs coated with different polymers

Previously, we have shown that NPs promote cross-presentation and require APCs autophagy [9]. In order to understand the reasons why the positive charged γFe_2_O_3_ NPs could help DCs cross-presentation, we analyzed the autophagy protein LC3 by western blot. We found that LC3-II formation was in a time-dependent manner and it required approximate 3 h to reach maximum. The γFe_2_O_3_/DMSA with negative charges induced the formation of LC3-II protein 1.5 h earlier than the γFe_2_O_3_/APTS NPs (Fig. 6). These results indicate that both negative and positive charged NPs could induce autophagy, and negative charged NPs induced autophagy more rapidly than positive charged NPs.

### OVA Absorption by NPs and Cytosolic Delivery

**Fig. 7:**
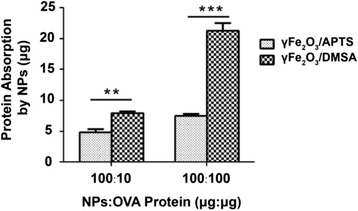
OVA protein absorbed by NPs

To further understand why γFe_2_O_3_ NPs coated with APTS could help DCs cross-presentation, we firstly examined the OVA protein absorption ability of NPs. There was a considerable difference between the two NPs and the similar result was detected between the two doses of OVA protein. Due to the different surface charges, the amount of OVA protein absorbed by γFe_2_O_3_/APTS NPs and γFe_2_O_3_/DMSA NPs (100 μg) were 4.87 and 7.98 μg in the 10 μg OVA group and 7.57 and 21.30 μg in the 100 μg OVA group, respectively (Fig. 7). There were significant differences in the two groups.

**Fig. 8:**
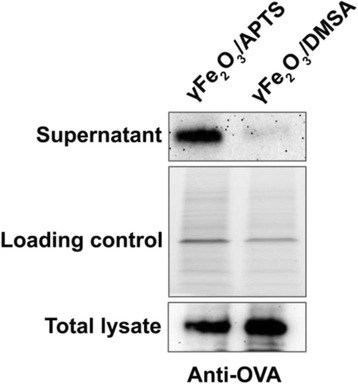
OVA expression in the cytosol and total lysate of DC

Negative charged γFe_2_O_3_ NPs can absorb more antigen protein than positive charged γFe_2_O_3_ NPs, but they were less efficiently cross-presented than positive charged NPs. We postulate that cytosolic delivery of OVA is influenced by surface charge variability. We collected cytosol for Western Blot analysis from DCs cocultured with OVA and opposite charged γFe_2_O_3_ NPs for 30 min after PFO was added. There was an obvious signal of OVA in the γFe_2_O_3_/APTS NPs group, which means OVA protein was released from the cytosol. By contrast, there was no signal in the γFe_2_O_3_/DMSA NPs group. Without PFO treatment, DC loaded with NPs coated with OVA did not release any proteins into supernatants (Fig. 8).

## Discussion

The synthetic SPIO NPs have been used as a cell labeling and imaging contrasting agent safely and effectively in our previous studies [12, 13]. The crystalline core of these particles is composed of γFe_2_O_3_ NPs and carry a positive surface charge of 20.9 mV. These particles have an average diameter of 8.7 nm and a hydrodynamic size of 92 nm [19]. Two opposite charged coating, APTS (positive) and DMSA (negative), were selected in this study to investigate the influence of surface charge on the antigen delivery and antigenic cross-presentation by DCs. It has been shown by zeta potential measurement that γFe_2_O_3_/DMSA particles have a high negative potential, while γFe_2_O_3_/APTS particles have a (low/high) positive potential [15]. These different charges may be attributed to the different functional groups, such as the carboxyl and sulfylhydryl groups, which ionize in the physiochemical state. Furthermore, these particles will roll into the endocytosis transports process after they enter into the cells. We still do not know the delivery process after the charged particles are engulfed into the cells. And, the influence in the delivery systems of the APCs is not clear after the SPIO NPs coated with opposite charged polymers enters into the cells.

To answer these questions, the zeta potentials of different NPs coated with or without OVA were detected in various pH values. According to the IP of OVA protein (pH = 4.8), which is an acidic protein under physiological condition [20]. The potential will be positive when the pH is under 4.8 and negative above 4.8.

We attempted to determine whether γFe_2_O_3_ NPs coated with different charged coatings, APTS or DMSA, could be used as adjuvants to promote antigen cross-presentation by DCs. OVA protein was selected as models antigens. Mutu DCs, an immortalized murine DCs cell line, was used as APCs. Meanwhile murine B_3_Z cell line was chosen as the responder cells and these cells activation were detected by the assay of CPRG.

In our studies, we showed that OVA protein could be presented by murine DCs to CD8 positive T cells efficiently with the help of γFe_2_O_3_/APTS. On the contrary, even though the antigens could be brought into DCs by γFe_2_O_3_/DMSA, they failed to induce an effective T cell response (Fig. 2). These results suggest that the vessel charges around the NPs play an important role in the delivery of vaccines into APCs and also influence antigen cross-presentation, which optimizes the surface properties of NPs as being adjuvants. It has been reported that the DMSA modification may facilitate SPIO and gold nano-shells adhesion to the cells membrane and enhance the cellular uptake [21, 22]. However, we titrated the antigens and γFe_2_O_3_ NPs coated with APTS or DMSA at different concentrations of DCs cross-presentation and found that the response of T cells induced by γFe_2_O_3_/DMSA delivery antigens was much lower than that of γFe_2_O_3_/APTS when the concentrations of γFe_2_O_3_ NPs were between 10 to 300 μg/ml. Both kinds of particles express almost no T cell responses when the concentration of particles was below 3 μg/ml. Thus, we can draw the conclusion that positive charged γFe_2_O_3_ NPs could function as efficient adjuvant for antigen cross-presentation, but the γFe_2_O_3_ NPs with negative charged coating cannot despite of more proteins being brought into cells.

Based on previous reports, cytosolic antigens were degraded into peptides by the proteasome and then transported into the ER by the transporter TAP1 during antigen cross-presentation. Peptides loaded with MHC-I molecules were then released to the cell surface via the Golgi. DCs have the capacity to present peptides degraded from endogenous or exogenous antigens on MHC I molecules through cross-presentation. CD8 positive T cells are activated by DCs through antigen cross-presentation, an important mechanism for the development of cytotoxic T cell (CTL) responses against pathogens and tumors [23]. Among cross-presentation, one of the most important routes is the cytosolic pathway, in which the antigens are translocated from phago/endosome into the cytosol, where they are further processed by the proteasome and TAP1 transporter but not the lysosomal proteases [23].

To demonstrate whether the TAP1-transporters pathway is related to the antigen transmission for cross-presentation in the cytosol, the TAP1 knockout DCs was induced in our study. Compared with Mutu DCs, we found that there was almost no response after TAP1 was knocked out from the DCs (Fig. 3). Collected data showed the pathway of antigen delivery in DCs for cross-presentation activated by the positive charged NPs was TAP1 dependent. To further clarify the antigen delivery route in DCs, proteasome and lysosome inhibitors, velcade and NH_4_Cl, were adopted in these studies. In DC cross-presentation, NH_4_Cl did not block the T cells responses. However, velcade blocked it completely (Fig. 4). These data revealed that OVA protein can be presented by DCs to CD8^+^ T cells via the proteasome but not the lysosome. To further explain this question, location of γFe_2_O_3_ NPs with opposite charged polymers in DCs were examined by TEM. According to TEM, γFe_2_O_3_ NPs with positive charged APTS stayed in the early endosome or in the cytoplasm, while negative charged γFe_2_O_3_/DMSA was be found in the lysosome or autolysome covered by single or multi-layered membrane vacuoles (Fig. 5).

Since autophagy plays an important role in antigen cross-presentation in APCs, we studied the autophagic proteins LC3-I and LC3-II by Western Blot. We found that the expression of autophagic typical protein, LC3-II, in DCs cocultured with a negative charged polymer was much faster than that of positive charged polymer. Within a 6-hour time frame, we found that both negative and positive charged NPs expressed nearly the same amount of LC3-II. Therefore, we deduce that opposite charged NPs induce different levels of antigen cross-presentation and are not influenced by autophagy. It has been established between autophagy and innate or adaptive immunity that autophagy can regulate the intracellular killing of some bacteria and is also involved in the presentation of antigens through MHC-I and MHC-II molecules [24, 25]. From these results, we believe antigen proteins with negative charged NPs are swallowed into DCs, where they remain in the lysosome or autophagosome to induce more autophagy and release more cytokines (Fig. 6).

Even protein together with opposite charged NPs can be delivered through the proteasome, inducing autophagy and the inflammasome in DCs. In order to support our hypothesis, it is needed to demonstrate that the protein stayed in the DC’s cytosol. To prove this, OVA protein expression was examined in the cytosol of DCs by Western Blot. We used the membrane pore forming protein, PFO, which can perforate the cytomembrane and release the cytosolic proteins into the medium. If the OVA proteins cross the endosome membrane and translocated into the cytosol, it will be released by PFO and detected by western bolt. On the contrary, OVA protein will not be detected in PFO treated cell supernatant if it is sequestered in the lysosome, endosomes, or other compartments. We hypothesized that positive charged NPs promote cross-presentation by delivering more OVA into the cytosol. In Fig. 8, OVA protein did release from the cytoplasm of DCs cocultured with γFe_2_O_3_/APTS NPs after perforation by PFO. However, OVA protein was not found in cytosol of γFe_2_O_3_/DMSA NPs groups. Our collected data displayed that the antigen engulfed together with γFe_2_O_3_ NPs coated with positive charged polymer was located in the cytosol of DCs where it participated in antigen cross-presentation via the proteasome pathway. On the other hand, the antigen combined with γFe_2_O_3_ NPs coated with negative charged polymer did not exist in the cytosol of DCs. These results infer that the antigen with γFe_2_O_3_ NPs coated with the negative charged polymer sequesters in the compartments of DCs. Based on previous reports [15, 26], we speculated that γFe_2_O_3_ NPs coated with negative charged polymer will gather in lysosome or autophagosome of the DCs.

In summary, current studies prove that antigen cross-presentation of DCs can be enhanced by γFe_2_O_3_ NPs modified by positive charged molecules. These antigens were brought into DCs by γFe_2_O_3_/APTS and then cross-presented to T cells through the cytosolic pathway, which involved the proteasome and TAP1 transporter. However, the negative charged NPs inhibited the DCs functions by sequestering the antigen in the intracellular compartments and activating rapid autophagy. Overall, our results suggest that NPs modified with different chemistry exhibit diverse biological properties and differ in their adjuvant potentials. This will make us consider more comprehensively and carefully design of effective and safe adjuvants in the future.

## Conclusions

The positive charged particles can promote the antigen delivery in cytoplasm, which is beneficial for antigen cross-presentation of DCs and T-cell activation. NPs modified with different chemistries exhibit diverse biological properties and differ greatly in their adjuvant potentials.

## Abbreviations

APCs: Antigen presenting cells
APTS: 3-aminopropyltrimethoxysilane
BMDCs: Bone marrow derived dendritic cells
BSA: Bovine serum albumin
CPRG: Chlorophenol red-β-D-galactopyranoside
CTL: Cytotoxic T cell
DCs: Dendritic cells
DMSA: Meso-2, 3-dimercaptosuccinic acid
ER: Endoplasmic reticulum
MHC-I: Major histocompatibility complex class I
NPs: Nanoparticles
OVA: Ovalbumin
PBS: Phosphate buffer saline
PFO: Perfringolysin O
SPIO: Superparamagnetic iron oxide
TAP: Transport processing protein
TEM: Transmission electron microscope
